# “I’d been like freaking out the whole night”: exploring emotion regulation based on junior doctors’ narratives

**DOI:** 10.1007/s10459-017-9769-y

**Published:** 2017-03-17

**Authors:** Robert M. Lundin, Kiran Bashir, Alison Bullock, Camille E. Kostov, Karen L. Mattick, Charlotte E. Rees, Lynn V. Monrouxe

**Affiliations:** 1 School of Medicine, Cardiff University, Wales, UK; 2 School of Social Sciences, Cardiff University, Wales, UK; 3 Centre for Research in Professional Learning, Graduate School of Education, University of Exeter, England, UK; 4 Faculty of Medicine, Nursing and Health Sciences, Monash University, Melbourne, Australia; 5 Chang Gung Medical Education Research Centre, Chang Gung Memorial Hospital, Linkou, No.5, Fuxing St., Guishan District, Taoyuan City, 33305 Taiwan, ROC

**Keywords:** Audio diaries, Emotion regulation, ER, Gross, Junior doctors, Medical education, Preparedness for practice, Interns, Transitions

## Abstract

The importance of emotions within medical practice is well documented. Research suggests that how clinicians deal with negative emotions can affect clinical decision-making, health service delivery, clinician well-being, attentiveness to patient care and patient satisfaction. Previous research has identified the transition from student to junior doctor (intern) as a particularly challenging time. While many studies have highlighted the presence of emotions during this transition, how junior doctors manage emotions has rarely been considered. We conducted a secondary analysis of narrative data in which 34 junior doctors, within a few months of transitioning into practice, talked about situations for which they felt prepared or unprepared for practice (preparedness narratives) through audio diaries and interviews. We examined these data deductively (using Gross’ theory of emotion regulation: ER) and inductively to answer the following research questions: (RQ1) what ER strategies do junior doctors describe in their preparedness narratives? and (RQ2) at what point in the clinical situation are these strategies narrated? We identified 406 personal incident narratives: 243 (60%) contained negative emotion, with 86 (21%) also containing ER. Overall, we identified 137 ER strategies, occurring prior to (n = 29, 21%), during (n = 74, 54%) and after (n = 34, 25%) the situation. Although Gross’ theory captured many of the ER strategies used by junior doctors, we identify further ways in which this model can be adapted to fully capture the range of ER strategies participants employed. Further, from our analysis, we believe that raising medical students’ awareness of how they can handle stressful situations might help smooth the transition to becoming a doctor and be important for later practice.

## Introduction

Emotions comprise a physiological arousal and cognitive appraisal of situations, thus are ever-present in day-to-day medical practice (Redinbaugh et al. 2003; Satterfield and Hughes 2007; Saunderson and Ridsdale 1999; Vegni et al. 2001). As well as dealing with the emotions of others around them, physicians have their own emotions to manage.

### The impact of emotions

Rather than classifying all negative emotions as ‘bad’ and positive emotions as ‘good’, research suggests that the impact of both positive and negative emotions depends upon the specific context in which they occur (Ford and Mauss 2015). Thus, positive emotions (e.g. happiness, joyfulness, contentedness) can enable a physician to form strong doctor-patient relationships fundamental for history taking and clinical diagnosis (McNaughton 2013). However, there are times when up-regulating positive emotions can be problematic (Tamir and Ford 2012). For example, when anticipating altercation, it can be more beneficial to up-regulate negative behaviours (Ford and Mauss 2015; Tamir and Ford 2012). However, there is also strong evidence to suggest that negative emotions (i.e. feelings such as sadness, anxiety, fear and anger) can have significant adverse effects on some situations. For example, doctors’ negative emotions can be deleterious on their clinical decision-making and on health service delivery (Resnick 2012). Indeed, negative emotions can leave a clinician feeling anxious and uncertain of their own abilities (McNaughton 2013) and can lead to substantial illness and even suicide (Beyond Blue 2013; GMC 2014). Therefore, doctors’ abilities to regulate emotions can have important consequences for their attentiveness to patient care, patient satisfaction and for their own well-being (Kafetsios et al. 2014; Ogundipe et al. 2014; Sablik et al. 2013; Wu et al. 2014).

In terms of clinician well-being, emotional exhaustion has been shown to be one of the main symptoms of burnout (Maslach and Jackson 1981). Research suggests that the scale and impact of burnout and mental illness are greater in the medical profession than other professions (Figley et al. 2013). Using the Maslach Burnout Inventory, Shanafelt et al. (2012) found that almost half of the 7288 US physicians in their study reported at least one symptom of burnout. Physicians were more likely to report such symptoms and be dissatisfied with their work-life balance than the general US population. They also found considerable differences in burnout across specialties: the highest rates were evident amongst those at the front line of patient care (family medicine, general internal medicine and emergency medicine). Furthermore, rates of burnout and drop-out from medical school due to emotional distress are on the increase (Arulampalam et al. 2007; Ogundipe et al. 2014).

There is growing evidence to suggest that experiences during early postgraduate medical education can be particularly emotionally intense (Austenfeld et al. 2006; Redinbaugh et al. 2003). The transition from medical school to doctor is known to be a particularly demanding time, emotionally and physically (Monrouxe et al. 2014), with many factors including lack of support and long working hours impacting on well-being (Hurst et al. 2013; Markwell and Wainer 2009). Junior doctors (especially those in their first few months of practice) are at the bottom of the medical hierarchy in the clinical setting (Rees et al. 2007). As such, when they face situations that challenge their emotional strength, they sometimes struggle to find opportunities to openly express their emotions (Austenfeld et al. 2006).

## Emotions and junior doctors

There is surprisingly little evidence concerning the strategies that doctors (and particularly junior doctors within their first few months of practice) use to handle emotions associated with clinical experiences. What we do know is that doctors use a range of strategies to manage emotions including rationalising them, ruminating on them, talking about them, avoiding them, self-blame suppression and social support from colleagues, friends and family (Cherry et al. 2014; Figley et al. 2013; Laurent et al. 2014; Paice et al. 2002; Redinbaugh et al. 2003; Satterfield and Becerra 2010). However, these studies lack the use of any theoretical or conceptual framework. Furthermore, there is a paucity of research examining ER strategies used by junior doctors during early clinical practice. A greater understanding of this issue within a strong theoretical framework carries numerous potential benefits for doctors through shedding light onto potential support or educational initiatives. Ultimately this understanding can be used to facilitate the development of support mechanisms for doctors in relation to the emotional reality of their work, with the aim of reducing mental illness and encouraging them to stay in the profession (McNaughton 2013; Satterfield and Hughes 2007; Wu et al. 2014).

## Emotion regulation (ER): theoretical framing

While several theories have explored ER previously (e.g. Cicchetti et al. 1995; Eisenberg 2000; Kobak et al. 1993), we draw on Gross’ (Gross 1998; Gross and Thompson 2006) emotion regulation process model because it is a well-theorised and tested model and has been frequently used in the context of understanding, managing or coping with emotions when work roles demand an outward appearance of emotional control within the workplace (Grandey 2000). Epistemologically, Gross’ model originates within a social cognitive perspective that focuses on how individuals perceive, interpret and regulate information within social or cultural contexts. Essentially, emotion regulation (ER) comprises the processes by which we manage our emotions in terms of when we have them, how we experience them and how express them (Gross 1998; Gross and Thompson 2006).

Gross (Gross 1998; Gross and Thompson 2006) explains how ER can occur prior to the onset of emotion (antecedent-focused) and following emotional arousal (response-focused) by drawing on the “modal model” of emotion: specifically highlighting the *situation*-*attention*-*appraisal*-*response* sequence within an emotion-generative process. Thus, a ‘psychologically relevant’ situation occurs. This can be either in the form of an actual situation (e.g. a bleep going off during the night-shift) or in the form of an internal representation of a situation (e.g. I thought my bleep might go off during my night-shift). Such a situation is then attended to and appraised in-the-moment, which subsequently affects our experiential, behavioural and physiological response to the emotion experienced.

Emotion regulation (ER) is therefore a behavioural response: “the activation of a goal to influence the emotion trajectory” (Gross et al. 2011). These goals (or ends) require regulatory ‘strategies’ (or means to achieve the ends) that might either be under deliberate control or might be implicit processes. Gross’ (Gross 1998; Gross and Thompson 2006) process model of ER draws on this modal model of emotion-generation, specifically considering the on-going choices people make as they regulate their emotions occurring prior to, or after the *emotion*-*generative moment*. Thus some types of ER strategies are implemented in order to avoid an emotion, while others are employed to manage an existing emotion. As such, his theoretical model focuses on an interrelated series of processes unfolding chronologically across the emotion arousal-response event. Here, the term *event* refers to the temporal aspects from *pre*- *to post*-*emotion*-*generative moment* that occurs at least once, and sometimes multiple times, within a ‘psychologically relevant’ situation.

Thus, prior to any given situation we often have a choice (to attend or not, to go alone or with others, to wear certain clothes and so on). If this choice affects the potential emotional impact of the situation in that it has the possibility of avoiding an *emotion*-*generative moment*, then this decision can be classified as ***situation selection***. Situations can also be altered to modify the emotional impact they may have (***situation modification***). During the situation itself, we can also decide which aspects to attend to—which might alter our emotional response to it. When we do this to shift the emotional impact of any part of the situation this shift is called ***attentional deployment***. We can also opt to select one of many meanings to a given situation. Again, when this selection is made to shift the emotion impact that the meaning had, Gross calls this regulatory strategy ***cognitive change***. Finally, once the emotional response tendencies have begun (i.e. the emotion itself) we can change the emotional response itself by either up-regulating or down-regulating it. ***Response modulation*** is the term that is used for the process of altering one or more response tendencies upon emotion elicitation. It should be noted that some regulatory strategies are not restricted temporally and so can occur prior to, during or following the situation itself (e.g. response modulation), whereas others are temporally fixed (e.g. situation selection). Table 1 summarises Gross’ (Gross 1998; Gross and Thompson 2006) process model of emotion regulation further by specifying the different ways in which these strategies manifest themselves (e.g. through distraction, avoidance, rumination etc.).

**Table 1:** Summary of Gross’ (Gross 1998; Gross and Thompson 2006) process model of emotion regulation

ER strategy	Situation selection	Situation modification	Attention deployment	Cognitive change	Response modulation
Description	Choosing a situation based on the expected emotional outcome	Altering the situation in some way to change the emotional outcome	Focussing on specific aspects of the situation, including the emotional aspect (*rumination*), seeking new information and using existing knowledge (*information*) and on non-emotional aspects (*distraction*)	Seeking to purposively shift one’s thinking to alter emotions (*reappraisal*)	Regulating one’s emotional response by removing oneself from the situation (*physical*), expressing one’s emotions outwardly (e.g. crying) or inwardly (e.g. *suppression*, *moping*) and sharing emotions or situation with another

ER by itself is neither *good* nor *bad* and the effects of regulating emotions can be contradictory, depending on the context. For example, dampening negative emotions may help a doctor to work efficiently in stressful situations but might also quash emotions associated with empathy (Gross and Thompson 2006). A worked example might help in understanding this model (see Box 1):

**Box 1:** First night shift as a junior doctor

Sandra is on her first night shift as a junior doctor. During the shift, Sandra’s bleep goes off and she is asked to see a patient whose clinical picture is deteriorating rapidly. To manage this potentially terrifying situation, Sandra has several options. She might try to avoid the situation altogether [possibly by not answering her bleep: *Situation Selection*] or she might ask another junior doctor to accompany her when she goes to examine the patient [*Situation Modification*]. She might add to her emotional state by concentrating on her anxiety [*Attention Deployment:* rumination], or focus on what she might do on arrival to the scene to assure support [*Attention Deployment:* distraction from emotion]. Alternatively, she might fight her anxiety by reassuring herself that she is well prepared [*Cognitive Change:* reappraisal]. When Sandra gets home after the shift, however, she continues to feel uneasy about that night shift’s unfolding events. One way of dealing with this would be to suppress her emotions and withdraw into herself [*Response Modulation:* suppression]. Alternatively, she could talk things through with her partner or a peer, sharing the events and her anxieties [*Response Modulation*: outward expression].	

Gross (2015) recently extended his model to address criticisms that the original model only identifies single, as opposed to multiple, strategies to be employed at any one time (Aldao and Nolen-Hoeksema 2013; DeSteno et al. 2013; Gross 2001; Kafetsios et al. 2012; Sheppes and Meiran 2007). Therefore, the revised model now accounts for the multiple ways in which decisions made at each time point can impact on a situation and the emotions elicited, along with the identification of three stages of an ER cycle: perception (or identification), valuation (or selection) and action (or implementation) (Gross 2015).

ER studies outside the medical context have typically looked for generalised patterns of ER use by exploring differences in gender, context, the impact of timing and the effectiveness of different strategies. These studies have found that women are more likely to report engaging in ER strategies than men, and are also more likely to report ruminating and seeking emotional support (Aldao and Nolen-Hoeksema 2012; Tamres et al. 2002). Stressful environments and the intensity of emotional stimuli have been shown to impact on individuals’ abilities to successfully regulate their emotions (Cheng 2003; Raio et al. 2013; Sheppes et al. 2011). Other research has proposed that regulation of emotion prior to an emotional response tendency is more successful than post-emotion regulation (e.g. suppressing the emotion as it emerges), which can decrease the expression of emotion but not the experienced emotion, and it can also impair memory (Gross 2001). In addition to demographic, environmental and temporal factors, certain strategies have been found to be more successful than others. For example, reappraisal and problem-solving are thought to be more successful than rumination and suppression, which have been shown to be correlated with psychopathologies (Aldao and Nolen-Hoeksema 2010; Gross and John 2003).

### Study aims and research questions

The aim of this study is to explore if, and how, newly graduated doctors narrate ER in their stories of preparedness and unpreparedness for their work (henceforth called their preparedness narratives) and whether Gross’ theory adequately captures the range of ER strategies as narrated in stories of actual situations. Through a secondary analysis of junior doctors’ preparedness narratives, the following research questions (RQs) were explored:

- RQ1: What ER strategies do junior doctors describe when narrating negative emotions in their preparedness narratives?
- RQ2: At what point in the clinical situation do junior doctors narrate ER strategies as a response to negative emotions?

Although, the concept of emotion regulation can be employed with regards to both positive and negative emotions, here we focus solely on the narration of negative emotion by junior doctors. Indeed, as Folkman and Moskowitz (2003) highlight, the common concern in everyday clinical contexts is the regulation of distress, rather than modifying or maintaining pleasant experiences, and this is even more the case for junior doctors narrating their relative preparedness for practice.

## Methods

### Design

This study comprises a secondary analysis of interview and audio diary data collected as part of a previous study examining junior doctors’ preparedness for practice (Monrouxe et al. 2014). This multi-site qualitative project collected personal incident narratives (PINs) from junior doctors across four UK sites (Wales, England, Scotland, Northern Ireland). Rather than asking about attitudes or beliefs, we employed a narrative approach for both study methods: audio diaries and interviews. The audio diary method of data collection comprises an initial group interview phase followed by audio diaries recorded over a set period of time, culminating in a further group interview phase at the end, or 6-monthly if over a longer period (Monrouxe 2009a, b). Narratives encourage participants to share their experiences relating to specific preparedness situations, thus providing access to participants’ lived experiences (Riessman 2008). Audio diaries are a unique tool for exploring how individuals make sense of specific events *in*-*the*-*moment*. The freedom that comes with personally recording narratives soon after the experience has provided powerful insights into junior doctors’ experiences, including the immediacy of emotional aspects (Monrouxe 2009a). For the junior doctors’ audio diaries, we simply asked them to: “tell us about a situation for which you felt prepared, and one for which you felt unprepared”. No other instructions were given to participants (e.g. they were not required to specify the time duration between the situation narrated and recording their diary, although participants sometimes volunteered this information). These audio diary entries allowed the newly qualified doctors to narrate difficult situations in this period and how they responded to them. We asked participants to record their audio diaries regularly, ideally on a weekly basis. If participants missed a week we established an unobtrusive method of reminding them (email or text message reminders depending on personal preference).

### Participants

Purposive sampling was used to achieve a maximum-variation cross-sectional sample. Following University, Medical School and/or Hospital Trust ethics committee and site-specific approvals at all 4 study sites, multiple methods were used to recruit participants through: emails, notices on virtual learning environments and notice boards, snowballing through organisations (e.g. British Medical Association junior doctor committee) and face-to-face recruitment during teaching events. Prospective participants received an information sheet and consent form, and were contacted to arrange an interview for the wider study, during which they were given the option to ‘opt-into’ the audio diary study (Monrouxe et al. 2014).

The 34 junior doctors participating in the wider study (Monrouxe et al. 2014) took part in individual or group interviews (depending on participant preference) between November 2013 and February 2014. Participants (18 male, 16 female) were between 23 and 40 years of age (80% between 23–25 years). Of these, 26 also went on to participate in the longitudinal audio diary study (averaging 3 months duration). These comprised equal numbers of males and females, between 25–29 years old. Nineteen of these 26 then participated in exit interviews (individual or group, as before) at the end of the audio diary phase. All participants in the study were in the early stages of their first year of medical practice after medical school.

### Primary analysis

An initial Framework Analysis (Ritchie and Spencer 1994) was developed by 10 researchers on the wider study examining preparedness for practice following the transition from medical student to junior doctor. Upon completion of the primary analysis it was identified that (a) being prepared for practice included the concept of emotional preparedness and being able to deal with one’s own negative emotions and that (b) junior doctors frequently felt unprepared for their own negative emotional responses. This initial analysis led us to decide to examine the strategies junior doctors narrated around how they regulated their emotions. A new group of researchers (RML, KB, CK) were recruited for a literature review and to identify suitable frameworks for analysis. This work led to a secondary analysis of the audio diaries focusing on emotion regulation strategies narrated by drawing on Gross’ theory of emotion regulation.

### Secondary data analysis

There were 254 audio diary entries (range 1–15 per participant) comprising 18:17:28 (hh:mm:ss) of audio data with 33 individual/group interviews (comprising 23:10:59 of audio data) in this data set. All data were transcribed verbatim, anonymised and entered into ATLAS.ti qualitative software for analysis. Unique identifiers were assigned to each participant to enable us to identify gender [F, M], data collection method [AD, INT] and participant number [1–34].

Our coding of the data was developed both deductively, based upon Gross’ model of emotion regulation (Gross 1998; Gross and Thompson 2006), and inductively from our data to give a single coding framework of the emotional regulation strategies narrated. This secondary analysis of data therefore differed from our original inductive thematic analysis. We were well placed to attend to the issue of ‘fit’ and ‘context’ (Hammersley 2010). The notion of fit refers to the difference between studies in which researchers interview key participants to generate data for answering specific research questions, verses those in which researchers use existing data collected for another purpose to address their research questions. The issue of context refers to the amount of contextual knowledge a researcher has when undertaking secondary analysis on existing data. This is important because data are not acontextual, rather they not only comprise what is recorded as data, but also include the “implicit understandings and memories of what they have seen, heard, and felt” (Hammersley 2010; point 3.4) during the process of data collection. Given that our secondary analysis followed on from our own identification of participants’ lack of preparedness in this area, and that the core research team from the original study led the analysis, this secondary analysis can be seen as an extension of the original purpose (rather than a re-use of data acontextually: Hammersley 2010). Problems of fit and context were thus minimal.

All researchers met online and face-to-face over several weeks to develop the framework for coding being mindful to represent the data as we saw it, rather than shoe-horning data into a priori categories. This was undertaken by: (1) reading and discussing a variety of aspects of Gross’ model and defining them for coding consistency; (2) reading and discussing a sub-set of the narratives, looking for relative ‘fit’ with the model, including aspects that were missing, or different, from it; (3) documenting the outline and definitions of all codes in the proposed framework (including those derived from Gross’ model and those that extended the model), along with a crib-sheet for coders; and (4) talking through a range of narratives to achieve clarity and consensus on classifications of specific codes to narrative sections.

Three researchers (KB, CK, RML) undertook the coding with the supervision of LVM. The coding from the secondary analysis was added to the original coded data in ATLAS.ti software for completeness. For example, as the data had already been coded according to whether the narrative depicted the narrator as prepared or unprepared for specific areas of practice, we could examine this alongside ER. In terms of coding for preparedness, narratives were often complex with elements of both preparedness and unpreparedness. We classified all narratives as a whole depending on how the narrator constructed the situation (narratives frequently began with an evaluation such as ‘a time when I felt prepared/unprepared…’). When narratives were used as a general illustrative example of generalised issues, we coded them as ‘unspecified’.

All narratives were read and listened to concurrently (for extra-linguistic information regarding emotion such as tone and volume of voice, hesitations and laughter-talk: Rees and Monrouxe 2013). They were then coded according to the presence of emotion and/or ER as (1) No negative emotion narrated, (2) Negative emotion narrated or emotional tone used (i.e. the narrative was said with an emotional tone, including the use of laughter for coping, but no specific emotion was narrated) without ER, (3) Negative emotion, or emotional tone, with ER. All data were double-checked by the researchers for consistency and any disagreements in coding were discussed and resolved with LVM and EH (see acknowledgements). Finally, LVM checked the coding of all PINs for completeness. A frequency table for the most commonly used words was created using the ATLAS.ti software to examine the content of the emotional talk. Although the data collected were longitudinal, we do not examine these developmentally.

## Results

We identified 406 distinct personal incident narratives (PINs) from the combined interview and audio diary narrative data. Audio diaries typically contained a single narrative, although some contained two. Table 2 outlines the presence of ER strategies and how they relate to the preparedness narratives.

**Table 2:** Total (%) of PINs classified by emotion, ER and preparedness situation (total n = 406)

Narratives	Total	Prepared	Unprepared
No narrated negative emotion	163 (40.1%)	106 (59.5%)	57 (25.0%)
Negative emotion, no regulation	157 (38.7%)	48 (27.0%)	109 (47.8%)
Negative emotion with regulation	86 (21.2%)	24 (13.5%)	62 (27.2%)
Totals	n = 406	n = 178	n = 228

In terms of the negative emotion content, the most commonly used words across 243 narratives with emotion were worry/worried (n = 69), scary/scared/scariest (n = 32), stressful/stress(ed) (n = 47), fear (n = 32), upset (n = 27), horrible (n = 24), daunting (n = 20) and angry (19). Of the 86 narratives that contained ER, 62 (72.5%) contained single, rather than multiple regulation strategies (27.5%). Overall, our participants narrated 137 ER strategies occurring prior to (n = 29, 21.2%), during (n = 74, 54%) and after (n = 34, 24.8%) the situation.

In order to answer our research questions, we outline the different ways in which participants narrated ER strategies prior to, during and after their preparedness situations. In the excerpts we underline emotional words, or where emotional tone was narrated, with talk relating to the ER strategy marked in bold [and identified in square brackets]. Three dots … indicate where sections of the data are removed for brevity, with double brackets providing additional information, for example ((laughs)). Finally, in the UK (where this study was conducted) junior doctors are known as Foundation Year 1 (or F1) doctors, we have changed references to F1s to read ‘junior doctors’ for the non-UK reader.

### Emotion regulation prior to situations

Participants narrated four main types of ER strategies used in anticipation of situations (n = 29, 21.2%) that broadly align to the categories described by Gross: Situation Modification [SM], Situation Selection [SS], Cognitive Change [CC] and Attention Deployment [AD].

#### Situation modification [SM] and selection [SS] prior to the situation

We have two examples where participants specifically ‘selected’ the situation to avoid a difficult emotional outcome. Both of these narratives involved participants who felt emotionally unable or unwilling to attend to the patient so passed them onto someone else. For example, on realising that she knew the patient and the patient’s family, one participant alerted her senior and the nurses present:

A former colleague’s parent of mine was admitted as an emergency… Unfortunately they had suddenly deteriorated and were admitted as an emergency… their prognosis was very, very poor. I had mentioned to the SHO ((senior house officer, junior doctor with more than one year’s experience)) my personal involvement with this patient so that he was aware that I was finding it quite difficult, **I just thought it best to make that that open so that people were aware** [*SM*]. Unfortunately over the course of the night I was called to see this patient - they were opiotoxic ((acute condition due to an excess of opioids)) something I hadn’t particularly dealt with before. I was quite sad about the situation **so upon arriving to the ward I discussed it with the nurses… we thought it was maybe best to get the SHO down… just to benefit everyone really to have someone more senior taking charge** [*SM*] [FAD170]

Interestingly, although her first modification strategy (informing the SHO) was narrated as being solely for the benefit of herself and to regulate her emotions, her second attempt at modification was narrated as benefiting the patient also. Indeed, this dual purpose of mitigating one’s own emotional distress and benefitting patient care were commonly narrated. For example, participants sometimes modified the situation by calling for help from their seniors, the wider team or their peers to assist when negative emotions arose due to lack of competence:

I was asked to do an incision and drainage of an abscess on the back of a lady’s neck, but the way they said it was ‘*can you go and do it please*’… there was no ‘*have you done this before?*’ or ‘*do you know what you’re doing, do you know how to do it?*’… I had seen one before but I’ve not actually just done one on my own before… and it was quite scary… there’s part of me that just wants to think ‘*right, I know what it is, I just need to get on and do it*’ but I thought ‘*I’m not 100% confident* *in doing that on my own,* *I don’t feel like I can just go ahead* *and do that that procedure without any further guidance*’ **so I had to stop and say** ***‘I could do with someone watching over me doing this again’ rather than it just be me going off and doing it*** [*SM*] [MAD16]

#### Cognitive change [CC] prior to the situation

In addition to calling for support, some participants narrated how they reframed their thinking in order to overcome their initial emotional responses to an anticipated situation. This was done using the emotional support of seniors and peers, receiving esteem support such as encouragement from others in order to change their mind-set (e.g. one participant felt nervous about suturing and was encouraged and supported by a senior to complete the task) and through personal reappraisal of their own abilities:

sometimes you kind of think ‘*this is a senior nurse who’s been cannulating day*-*in and day*-*out since they started, they probably have far more experience than you do*’ and… suddenly when they can’t do something, the next step is to ask you, it’s quite a lot of pressure because… the patient’s already been stabbed a few times and that the nurses have been having a go and they all then look to you, and you really have to perform, and you can’t show the patient that actually you’re worried, you can’t show the patient that there is a chance you’re going to miss it you- you just have to accept that you are the next step in trying to get this cannula in, **and I actually felt quite confident, I feel like, I was well trained at medical school**…[*CC:* reappraisal] [FAD08]

#### Attention deployment [AD] prior to the situation

Some participants used information as a way of reducing their anxiety of an impending situation. The use of information at this point in time appeared to be more for the benefit of managing the emotional outcome for the junior doctor rather than for the patient. This involved reframing the situation away from the emotion, instead focussing on something else, usually something more practical. For example, following one participant’s medical school experiences with ‘harsh’ doctors, she managed the ‘daunting’ prospect of interacting with a senior from outside her team by reading up on the patient thoroughly and preparing a summary prior to contacting them:

I wasn’t very good at speaking to seniors from another team over the phone, I was quite worried about doing it because it can be quite harsh at times… **so I read the patient’s notes from when they were admitted and wrote on a separate piece of paper a short summary of everything that had happened, including the results from the diagnostic tap, the patient’s blood results and stuff like that** [*AD:* information use], so that when I bleeped the registrar ((more senior trainee doctor)) on their respiratory team I could quickly reel off what had happened to the patient since she’d come into the hospital**…** because it can be quite a daunting situation- especially if you don’t know the answer you end up looking stupid [FAD01]

It was not just communicating with doctors that drove participants to seek out information prior to situations. Knowing they might be called upon to answer patients’ questions also motivated them to reduce anxiety and stress through AD. The following example is particularly interesting as the participant draws on the metaphor of doctor-patient relationship as war (Rees et al. 2007), as he ‘armed’ himself with information before getting a ‘good grilling’:

it’s quite a- quite a high stress environment…a sixty-five-year-old lady turned up to the MDU ((Medical Defence Union)) with a referral from her GP and he’d queried Bell’s Palsy… I’ve only ever seen one or two in my life before so I didn’t exactly know… what the answers to any questions that the patient should ask me… I was put in quite a difficult position because the consultant was busy… I needed to make sure I knew exactly what I was talking about without giving them ((*patient and family*)) false information. There’s a brilliant app which I subscribed through the ((*names university*)) called the ((*name*)) which basically allows you to access a lot of the Oxford Handbook material… you’ve got all the information at your fingertips… so after reading up… **I kind of armed myself with that information before going into the consultation** [*AD:* information use] … patient’s daughter … she gave me quite a good grilling [MAD19]

What is interesting about the use of information prior to the situation is that while it is technically within Gross’ model a form of AD, the effect it has is one of Cognitive Change: participants often mentioned feeling less emotional and more able to undertake the sometimes daunting tasks that lay ahead of them. This is akin to Gross’ notion of intellectualisation (Gross and Thompson 2006). Another common type of AD employed prior to the situation was rumination. However, unlike the use of information, rumination led to feelings of apprehension, anxiety and stress. This was particularly evident in the audio diaries as participants narrated the impending events of the next few days.

### Emotion regulation during situations

The majority of ER strategies were identified during the situations (n = 74, 54%): these were classified as being Attention Deployment (AD), Cognitive Change (CC) and Response Modulation (RM).

#### Attention deployment [AD] during situations

In terms of AD, one common form of response was rumination. Rumination involves directing one’s attention to the negative aspects of the situation and the negative feelings associated with this. Participants talked a lot about how they worried during their shifts that treatment they had given was incorrect, checking and double-checking treatment doses and procedures. A few participants highlighted how their seniors appeared to take this more in their stride:

he seemed quite relaxed about the whole thing whereas I’d been like **freaking out the whole night** [*AD*: rumination] about ‘*why is this blood pressure still sitting low*’ [MAD26].

Some participants felt supported by this relaxed attitude, whereas others felt further anxiety, worrying that they might be slowing things down on the ward and be viewed by others as being difficult (further rumination about the consequences of their ‘worrying’). However, the fast pace of ward life often meant that rumination was a ‘time limited’ activity:

During the same shift… two patients on the same ward had died within 20 min of each other… the patient’s wife was not accepting that he had really gone… I felt apprehensive… I felt a bit anxious… [*AD*: rumination] A few minutes later the ‘phone rang and I answered… it was an elderly man enquiring how his brother, the recently deceased patient, was keeping. This totally caught me off guard. There was a silence which felt like an eternity to me as I struggled to dredge up from the back of my mind the breaking bad news training… ((*she gives details of the call*))… **I then had to go to another ward to attend to tasks there so I was no longer able to dwell upon what had just happened** [*AD:* distraction from emotion] [FAD11]

In a similar vein, participants talked about how they ‘just got on with it’ when they felt overwhelmed, anxious or nervous rather than ruminating on their emotional responses:

it was just a bit nerve-racking… so that was that was a bit nervous, **I sort of got on with it though** [*AD:* distraction from emotion] [FAD12].

As such they distracted their attentions away from general negative emotions about their own capabilities onto the job at hand.

#### Cognitive change [CC] during situations

Many times participants reported shifting their thoughts by reframing the situation as one that required them to act, rather than to react. This transpired in a variety of ways including through the use of clinical skills, and in particular the so-called ‘*fire*-*drills’*. Fire drills are essential emergency procedures that have been ‘drilled into’ medical students throughout their undergraduate training. For example, students are taught the ABCDE (Airway, Breathing, Circulation, Disability, Exposure) approach that has wide application for assessing and treating all clinical emergencies. Because students are repeatedly being taught and assessed on this, by the time they are junior doctors it is second nature to them:

this ((*ABCDE*)) has just really helped me in lots and lots of situations because especially when you start, and you get called to a situation… a patient is unstable or… a cardiac arrest call, immediately- your body fills with adrenaline and you kind of lose any logical ((laugh)) train of thought, so **having the ABCDE system is just a really effective way of not necessarily even finding out what’s wrong with the patient… if you can’t think of anything else to do**, or, you know, your nerves just hit, **you then you just go through the ABCDE** [*CC:* clinical skills] [FAD06]

Using information to change how they felt about the situation was another common way participants dealt with negative feelings during the situation. Participants sought information from a variety of sources: talking with the patient and their families, other junior doctors, the nurses, the wider team including pharmacists, phoning around to get information about tests and using books and other sources on smartphones and tablets. In addition to information, participants drew on the emotional support of others in order to change the way they felt about the situation. For example, one participant talked about a ‘friendly SHO’ who helped her through a particularly difficult time and others talked about approachable nurses or consultants who appeared to have a calming effect, enabling participants to re-frame the situation from one of an emergency to one of a more routine ‘complication’:

I was like ‘ *this isn’t right’* so my registrar was just across the way, I just dashed into ((the ward)) and I was like ((name)) ‘*can I have a quick word*?’ and **he’s a really, really nice relaxed Scottish bloke, just really, really lovely… he’s very approachable** [*CC:* emotional support]… so he just walked in and he goes ‘*what’s up ((name))*?’ **put his arm around me** [*CC:* emotional support], walks in the cubicle and he goes ‘*oh what have you done*?’ ((laughter))… I was panicking quite a lot at this point, I was ‘*oh, oh she felt quite nauseous*’ and he goes ‘*did you give her Cyclizine*?’ I went ‘*yeah’* **he goes ‘** ***ah this can happen sometimes*** ***don’t worry about it*** **’** ((laughs))… I was just panicking so much and **he was just like so nice and relaxed about it and he goes ‘** ***what we’ll do*** **…**’ [*CC:* emotional support] [MAD19]

#### Response modulation [RM] during situations

When specific situations caused participants to experience negative emotions, if possible, participants sometimes distracted themselves from the situation itself. Such distraction often took the form of a physical action. For example, on finding that she had made a mistake, one participant reported leaving the scene to calm herself down:

**I took myself off the ward**… and just **took a minute in the staff room**… **pulled myself together** [*RM*: physical] [FAD10]

Other participants talked about walking away from stressful situations to prevent themselves from getting over-emotional or ‘stepping back’ metaphorically from the situation to calm themselves down. However, some participants narrated situations in which they responded outwardly to stressful situations on the wards, including raising their voices with patients, answering back to their consultants and crying:

at which point I was quite upset about the situation I was very fortunate that the nurses on the ward that evening were very experienced so I managed to discuss with them we came up with a plan we saw the family and we got it sorted. Unfortunately, **the emotional impact of this was quite high for me, I was very upset I was crying on the ward for quite a lengthy period of time** it was very difficult for me to deal with. I felt very isolated and left alone [*RM:* outwards] [FAD12]

Other participants talked about how they were angry inside, but managed to maintain a calm exterior for the sake of the patient.

### Emotion regulation following situations

ER strategies after the situations (n = 34, 24.8%) were all classified as Response Modulation, the majority of which comprised the sharing of emotions with others. The most common type of sharing of emotions following situations were with senior staff (particularly consultants) and peers, although the wider team were also involved (in particular nurses):

at one point my consultant sat me down and was like **‘** ***I’m going to have a conversation with you, as a father figure, now*** **’**… **he made me sit down and was like ‘** ***it’s going to be fine, we’ll move you over to the other side of the ward today so you can get away from it all and it will be ok*** **’** and he’s like **‘** ***don’t worry, I don’t doubt your ability, your resilience, anything like that, but there’s only so much one person can take and it’s all going to be fine*** **’** [*RM:* outwards - shared] [FINT27]

Indeed, across all phases of stressful situations, although participants also talked about sharing stressful situations with friends and family after their working day was over, it was the value of this senior support that was most commonly talked about. We also had two narratives in which participants talked about crying alone, away from others, as a form of emotional release:

I realise this is my 3rd entry this week but I thought I should make an entry about it. **I came home from work and** **cried today** [*RM:* outwards - not shared] and it’s the first time I’ve done that. I’m day 11 of 12 and I’m exhausted… we had 50 patients, my registrar was in theatre, both my SHOs were on call and so were not doing ward jobs and there were two junior doctors and two final year medical students, my bleep number is at the top of the list… it’s basically like being on call yourself and I was doing the junior doctor job and the SHO job and occasionally what felt like the registrar job for most of the day… your seniors see you like looking stressed its like ‘*oh they obviously just aren’t coping*’ but it’s having that responsibility, it’s a completely different kettle of fish… [FAD05]

## Discussion

Drawing on Gross’ theory of ER, we undertook a secondary analysis examining the range of emotion regulation strategies present in 406 narratives in which 34 junior doctors described situations for which they felt prepared (n = 178) or unprepared (n = 228) for practice during their first few months in post. Of these narratives, 157 (30.7%) contained negative emotion but no regulation strategies. From the 86 narratives that contained negative emotion accompanied by regulatory strategies three-quarters contained single, rather than multiple, strategies. A total of 137 instances of ER were identified, mainly narrated as occurring during, rather than before or after, the situation being narrated. We now summarise the strategies narrated at different times across the situations, then provide information on their relative frequency.

Specifically prior to situations, junior doctors narrated modifying the situation, or choosing not to attend, in order to emotionally manage the impending situation: often narrated as being for patients’ benefits, as well as their own. For example, modifications comprised calling for assistance from their seniors due to lack of competence, which is an appropriate response when faced with this kind of professionalism dilemma (Monrouxe and Rees 2012).

Both prior to and during situations, junior doctors narrated how they reframed their thinking about the situations and their capabilities, thus facilitating their actions rather than reactions. Interestingly, this cognitive change was achieved through a variety of means including relational (e.g. esteem support), practical (e.g. drawing on clinical skills training) and informational (e.g. phoning around, books, smartphones). Indeed, the use of mobile technology is reported to be on the increase amongst junior doctors due to their perceived increase in responsibility, alongside lack of knowledge and experience (Bullock et al. 2015). Junior doctors also narrated using similar resources to distract their attention away from their surging emotions: so-called *attention deployment*. The motivation here is not knowledge gain, rather it is to stop their emotions taking hold by focusing on something more practical and tangible. However, at times junior doctors narrated deploying their attentions towards these surging emotions, such as rumination, leading them to narrate anxiety and stress.

Finally, during and after situations, when emotional reactions were strong, junior doctors narrated a range of reactions to their emotions (*response modulation*) including mentally distracting themselves from the situation itself or ‘stepping back’, and sometimes physically removing themselves from the scene. However, some talked about other types of physical responses such as shouting and crying both during and after situations. Finally, talking about stressful situations with their seniors, friends and family comprised another response to emotional experiences.

Although our analysis is primarily qualitative, given this is the first study to examine ER strategies narrated by junior doctors, a consideration of the relative patterns of ER strategies is useful as a basis for further research (Sandelowski et al. 2009). The most commonly narrated ER strategies prior to situations were information use to achieve cognitive change and attention deployment. During the situations, the use of clinical skills accounted for the majority of all regulation activity both in terms of attention deployment and cognitive change. It was also the most frequently narrated ER strategy overall, followed by the use of information. This is of great importance as this is the first time that the use of clinical skills and information use have been highlighted as being productive devices specifically for the down-regulation of emotion in a healthcare setting. The main strategy narrated after situations comprised sharing the emotion or the situation with someone else (e.g. talking or crying with someone).

To our knowledge, only a single study has previously examined ER strategies employed in a healthcare setting, albeit not the main focus of the study (Kessler et al. 2012). Kessler et al. (2012) examined healthcare assistants’ (HCAs’) narratives of patient deaths and found that HCAs engage in cognitive change and attention deployment to manage their emotions during and after these situations. Thus attention deployment was used during the situation to distract their attention away from what was happening, although sometimes, as with our study, focusing on negative emotions was sometimes narrated (Kessler et al. 2012). However, these authors’ identification of cognitive change appeared to be attributed to shifts in thinking as a result of situations, to prevent long-term emotional distress, rather than during situations (as we have documented in this study).

### Strengths and limitations

The current work has several strengths and limitations. In terms of strengths, to our knowledge, this is the first study that has employed a theoretical framework (Gross 1998; Gross and Thompson 2006) to examine ER strategies among junior doctors. Another considerable strength is the use of unprompted narratives about preparedness and unpreparedness to explore emotions and ER. Participants were not specifically asked about emotions, yet more than half of the narratives contained emotion. In this way, we have been able to examine the use of ER strategies as narrated spontaneously. Thus the usual criticisms around self-reported data (e.g. that participants seek to present themselves in a positive light in terms of what they think is being examined) are mitigated to a certain degree. Additionally, the use of audio diaries is a strength in that it provides participants with the opportunity to record their narratives soon after the situation has occurred, in a private environment, facilitating the recall of details (including how they felt) that can be lost or dulled over time.

In terms of limitations, as discussed earlier, we did not explicitly ask junior doctors to tell us about their emotions and/or how these were managed. This has two obvious implications. Firstly, our analysis of regulatory strategies is based on researcher interpretations of the narrated situations, rather than participant declarations of regulation intent. Therefore it is possible that we may have over-interpreted some situations as being emotion regulation when they were not, and we may have missed other situations, emotions and strategies that occurred but were not narrated. Indeed, 40% of the narratives contained no negative emotion (either in tone or explicit emotion words). This is not surprising as 65% of narratives with no emotion primarily focussed on when junior doctors felt prepared. It is worth noting that only 25% of the *unprepared*–focussed narratives were devoid of any negative tone or emotion words. Furthermore, that the study was designed to focus on preparedness for practice, rather than emotion per se, means that it is possible that emotion was experienced in some incidents, but not narrated (due to participants thinking that emotions were irrelevant to the study). However, it is also possible that emotion *was* experienced but participants *suppressed* it, and that our research methods are unable to detect this strategy.

Other subcategories of ER identified by Gross (Gross 1998; Gross and Thompson 2006) not narrated in our data include downward social comparisons. Again, it is likely that our data collection methods were inadequate in terms of identifying this type of strategy. That some strategies are less narratable than others can be seen from the number of studies that state humour as an important strategy, yet no accounts of humour to regulate emotions in the situations described were narrated in our data (although sometimes humour and laughter were used in order to mitigate emotion experienced whilst narrating: Rees and Monrouxe 2013). Furthermore, it is likely that in-depth linguistic analyses would be required to investigate the use of humour in ER (Samson and Gross 2012; Wanzer et al. 2005; Wilkinson et al. 2007).

Where narratives were recorded close to situations, it is possible that some will have been narrated too soon to capture any ER that might have occurred later on. Furthermore, due to the immediacy of the audio diary method it could be that the very act of narrating their situations for this study comprised a regulatory strategy in itself (Monrouxe 2009b). It is also important to keep in mind that these are narratives of situations: recalled and subjectively narrated by participants, rather than recordings of the situations themselves and our analysis should be viewed in this light. Finally, we make limited claims to the transferability of the patterns we have found in our data to all junior doctors. Although data were collected across four UK countries, and we believe that some degree of transferability of findings to the wider UK cohort of junior doctors is a reasonable assumption, we make no claims regarding the ER strategies of junior doctors outside the UK. We therefore suggest that further explorations of ER strategies in this group of people across different countries, and in particular different cultures in which emotions might be variously expressed or inhibited, would enable us to ascertain whether similar patterns of strategies are present.

### Proposing a situation model of ER within clinical settings and future research

We now turn our attention to the question of how well Gross’ theory of ER captures self-reported ER strategies in narratives of situations experienced by junior doctors.

**Fig. 1:**
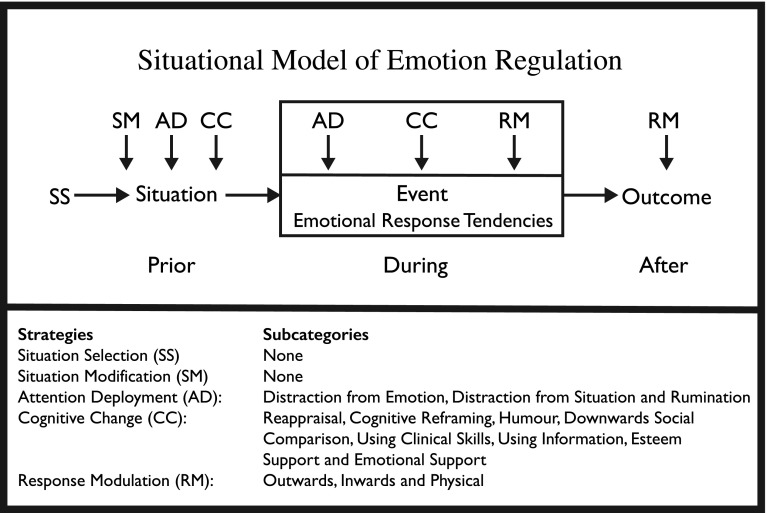
Situational model of emotion regulation The Situation Model of emotion regulation demonstrates the main categories of emotion regulation strategies that can be employed in relation to an experienced situation, such as those narrated by participants in this study, rather than the specific event of emotion arousal itself (i.e. as Gross defines it). It further allows for additional subcategories to be employed in addition to those suggested by Gross: SS, SM, AD (distraction from emotion, distraction from situation and rumination), CC (reappraisal, cognitive reframing, humour, downwards social comparison, using clinical skills, using information, esteem support and emotional support), and RM (outwards, inwards and physical)

As noted earlier, Gross’ model is an event-focussed model. However, the event in question comprises the emotion itself, rather than the broader pre-during-post situation-sequences that are typically storied by participants (Riessman 2008). Further, Gross' model has not been used previously in clinical healthcare settings. As such, we draw on Gross’ (Gross 1998; Gross and Thompson 2006) work to propose a situation model of ER within clinical settings (see Fig. 1). This model is not meant as a primary output of our results, but instead as the starting point for the refinement of a tool that can be used as a basis for future healthcare ER research. The model includes existing strategies determined by Gross with the addition of temporal aspects found in our study to enable researchers to build iteratively on findings, comparing across studies with different participants and settings. This situation model also allows ER strategies to be mapped onto situations where they naturally occur for the junior doctor, making it possible to compare the strategies used by different junior doctors in different situations and examine longitudinally how use of strategies change over time in an individual. Such an approach is necessary for us to determine the relative success of strategies employed in the clinical setting. Indeed, before we can make strong recommendations as to how emotion regulation should be taught to medical students, it is essential that we first determine which are more successful and why.

As can be seen in Fig. 1, our model includes the following: defining the situation temporally in terms of its antecedents, identifying the situation itself and identifying post situation behaviours (unlike Gross’ model that only attends to how we regulate our emotions prior to, or after the *emotion*-*generative moment*). Thus, our model can incorporate more than one emotion-generative moment during a single situation, thus capturing the full range of strategies utilised to manage emotions *over time*. This is an important factor as, for example, it will enable us to unpack temporal factors affecting the role of suppression as a successful strategy for dealing with high-pressure situations (Mitmansgruber et al. 2008) but also the role of suppression in psychopathology, leading to a decrease in positive and an increase in negative emotions (Aldao and Nolen-Hoeksema 2010; Gross and John 2003).

We name the response modulation subcategories ‘outwards’, ‘inwards’ and ‘physical’ to account for the range of strategies found within our participants’ narratives. Whilst not narrated in our data, we have added the use of laughter and humour based on other research undertaken with similar participant groups (Rees and Monrouxe 2013; Wanzer et al. 2005; Wear et al. 2006). Finally, we have added four types of social support to the categories of cognitive change and attention deployment (informational, emotional, esteem and physical: Schaefer et al. 1981) with physical support being renamed clinical skills to account for the clinical setting and the frequently narrated use of ‘fire drills’ (e.g. ABCDE) to regulate emotion. These changes are further supported by literature that identifies coping mechanisms typically employed by junior doctors (Levey 2001; Satterfield and Becerra 2010).

Further research specifically examining junior doctors’ ER strategies is now needed. Such research might begin by purposively inquiring about strategies employed in work settings through interviewing and audio diary methods, and later exploring perceived efficacy of these strategies across different participant demographics (e.g. gender, nationality, ethnicity). Such research would provide a more nuanced understanding of how junior doctors manage their emotions. Future studies could also examine the outcome of the situations, and the relative success of the ER strategies. This will be especially important for determining the success of specific strategies, the point at which they are employed, the use of multiple over single strategies, and ER in participants during transitional situations (e.g. the transition from medical student to junior doctor). Although early years of medical practice are of particular importance, it will also be interesting to explore how the presence (or absence) of ER strategies changes with time and clinical experience. Finally, we acknowledge that, due to our focus on a key transitional time and the issue of preparedness, feelings of uncertainty or anxiety might be over-emphasised, possibly resulting in a skewed understanding of the range and frequency of ER strategies employed by junior doctors. Exploring how junior doctors further into their training regulate other emotions—both negative and positive—is an essential next step.

### Implications for medical education

Considering the importance of ER for doctors, we believe that medical educators should find ways to raise students’ and trainees’ awareness of the strategies they might employ in stressful situations. Thus, task-focused skills that are typically taught to enable junior doctors to resolve clinical situations can also be explicitly taught as a way of managing their own emotion during difficult times.

For example, encouraging students and staff to share difficult experiences and consider how they manage emotions might promote the development of more effective ER strategies. Furthermore, through this sharing of their own strategies with others, it is likely that they might identify a wider range of automated or ‘hidden’ strategies for regulating emotion. Discovering further strategies through such exploration, along with developing our understanding through research endeavours, will help us understand further how doctors regulate their emotions. This may contribute positively to the health and welfare of future doctors, as well as patient satisfaction and quality of care.
